# From non-human to human primates: a translational approach to enhancing resection, safety, and indications in glioma surgery while preserving sensorimotor abilities

**DOI:** 10.3389/fnint.2025.1500636

**Published:** 2025-02-10

**Authors:** Matteo Gambaretti, Luca Viganò, Matteo Gallo, Giovanni Pratelli, Tommaso Sciortino, Lorenzo Gay, Marco Conti Nibali, Alberto Luigi Gallotti, Leonardo Tariciotti, Luca Mattioli, Lorenzo Bello, Gabriella Cerri, Marco Rossi

**Affiliations:** ^1^Neurosurgical Oncology Unit, IRCCS Ospedale Galeazzi Sant'Ambrogio, Milan, Italy; ^2^MoCA Laboratory, Department of Medical Biotechnology and Translational Medicine, Università degli Studi di Milano, Milan, Italy; ^3^IRCCS Ospedale Galeazzi-Sant’Ambrogio, Milan, Italy; ^4^Department of Oncology and Hemato-Oncology, Università degli Studi di Milano, Milan, Italy

**Keywords:** brain mapping, awake surgery, motor pathways, brain tumor, motor cognition, non-human primates, human primates, hand manipulation

## Abstract

Since the pivotal studies of neurophysiologists in the early 20th century, research on brain functions in non-human primates has provided valuable insights into the neural mechanisms subserving neurological function. By using data acquired on non-human primates as a reference, important progress in knowledge of the human brain and its functions has been achieved. The translational impact allowed by this scientific effort must be recognized in the implementation of the current surgical techniques particularly in support of the neurosurgical approach to brain tumors. In the surgical treatment of brain tumors, the ability to maximally extend the resection allows an improvement in overall survival, progression-free survival, and quality of life of patients. The main goal, and, at the same time, the main challenge, of oncological neurological surgery is to avoid permanent neurological deficit while reaching maximal resection, particularly when the tumor infiltrates the neural network subserving motor functions. Brain mapping techniques were developed using neurophysiological probes to identify the areas and tracts subserving sensorimotor function, ensuring their preservation during the resection. During the last 20 years, starting from the classical “Penfield” technique, brain mapping has been progressively implemented. Among the major advancements was the introduction of high-frequency direct electrical stimulation. Its refinement, along with the complementary use of low-frequency stimulation, allowed a further refinement of stimulation protocols. In this narrative review, we propose an analysis of the process through which the knowledge acquired through experiments on non-human primates influenced and changed the current approach to neurosurgical procedures. We then describe the main brain mapping techniques used in the resection of tumors located within sensorimotor circuits. We also detail how these techniques allowed the acquisition of new data on the properties of areas and tracts underlying sensorimotor control, in turn fostering the design of new tools to navigate within cortical and subcortical areas, that were before deemed to be “sacred and untouchable.”

## 1 Introduction

The role of surgery in the treatment of gliomas is crucial to relieve symptoms and determine histological and molecular diagnosis. The main oncological endpoints for surgery—namely progression-free survival, overall survival, and, in low-grade gliomas, malignant progression-free survival—greatly depend on the extent of surgical resection. Intrinsic brain tumors (in particular diffuse gliomas) are highly infiltrative, and frequently, the lesion harbors within the areas and/or pathways essential in neural networks subserving complex functions, such as sensorimotor control of dexterity, high motor skills, cognitive functions, and emotions. These areas are referred to as *essential structures*. A surgical lesion in these areas results in a neurological deficit, worsening the patient’s quality of life (Leonetti et al., 2021). Modern surgery aims at achieving the maximum tumor resection while preserving the patient’s functional integrity.

Accordingly, in the past 2 decades, the modern surgical approach has been changed from a traditional one, purely relying on anatomical and neuroimaging references, to a functional one, supported by the “brain mapping technique.” Brain mapping consists of the use of intraoperative neurophysiology protocols based on direct electrical stimulation (DES) that are applied to identify the *essential structures* based on their functional properties, to spare them from tumor resection. The efficacy of brain mapping is critically based on the knowledge of the neural circuits subserving neurological functions, implying both the anatomo-functional properties of the different neural structures (areas and tracts) and their specific role in the control of a given function. Consequently, the more refined the knowledge of the functional properties of the structures faced by the surgeon during resection, the higher the efficacy and resolution of the brain mapping approach in enhancing the resection borders to a total or even supra-total resection (Hervey-Jumper and Berger, 2016) preserving the sensorimotor, language, and high-order cognitive function integrity of the patient.

Resecting tumors involving the circuits subserving the highly refined human motor abilities is challenging, particularly when approaching tumors harboring within the corticospinal system’s areas/pathways that, when lesioned, result in permanent deficits due to their very low degree of postoperative plasticity.

The primary challenge in preserving motor functions—encompassing sensorimotor abilities and motor cognition—during resection lies in the deep understanding of the complexity of the sensorimotor circuits that govern voluntary movement. These circuits span various motor-related domains, from dexterity to praxis, and involve a wide range of cortical areas, extending far beyond the frontal lobe and ultimately engaging the association areas in the human brain (Simone et al., 2021; Fornia et al., 2024; Simone et al., 2020; Fornia et al., 2020b; Viganò et al., 2019; Fornia et al., 2022; Fornia et al., 2018).

Historically, procedures in patients with tumors growing in motor areas were performed in asleep conditions. This approach was based on the belief that motor skills could only be preserved by identifying areas responsive to stimulation with motor potentials (positive sites) and that the ability to track cortical and subcortical structures related to motor abilities was related solely to the protocol of stimulation, to be chosen between the available intraoperative protocols of stimulation, namely either the high-frequency (HF) or low-frequency (LF) DES (Szelényi et al., 2011; Taniguchi et al., 1993). A study published in 2014 by Bello et al. (2014) compared data obtained from the use of the two protocols, namely, the HF- and LF-DES, in the surgical removal of a large sample of gliomas involving the motor pathways, leading to the conclusion that it is the combination of the two techniques—rather than the choice between the two—defined by patient clinical history and tumor features shown by imaging, that increases the reliability of mapping, expands the number of patients who could benefit from surgery, optimizes the extent of resection, and decreases permanent morbidity. This study demonstrated that the clinical context significantly impacts the efficacy of brain mapping, highlighting the need for a tailored approach. It also emphasized the importance of further developing the technique based on the knowledge of the properties of the neural circuit to be preserved during resection, posing the starting point for the evolution of the technique over the past decade (Bello et al., 2014).

During the last 10 years, first, the specific anatomo-functional properties of the two distinct components of the corticospinal tract originating from the primary motor areas were described. This development enabled the creation of an advanced motor mapping protocol capable of differentiating between the various fibers (see Table 1). As a result, surgeons can now approach tumors located within the primary motor cortex (M1), which were previously considered unresectable (Rossi et al., 2021a).

**Table 1:** Comparison of the main neurophysiological properties of HF-DES and LF-DES.

	HF-DES	LF-DES
Stimulation frequency	250-500 Hz	50-60 Hz
Pulse form	Monophasic	Mono- or biphasic
Pulse direction		
Cortical	Anodal/positive	n/a
Subcortical	Cathodal/negative	
Duration of individual pulse phase	300-500 μs (standard) up to 800 μs (advanced)	500 μs
Number of pulses	5 (standard) 2-9 (advanced)	n/a
Common current intensity range and probes		
Asleep	5-15 mA Monopolar/Bipolar probe	7-16 mA Bipolar probe (always)
Awake	2-7 mA Monopolar/Bipolar probe	2-7 mA Bipolar probe (always)

The application of brain mapping techniques not only to M1 but also to non-primary motor areas (e.g., premotor and supplementary areas) and somatosensory areas has expanded our understanding of the sensorimotor circuits that control highly skilled voluntary movements (Fornia et al., 2024; Simone et al., 2020; Fornia et al., 2020b; Fornia et al., 2022; Viganò et al., 2022; Rossi et al., 2019). This progress was facilitated by the introduction of novel intraoperative tasks performed in awake conditions. These tasks are particularly critical for regions that lack a direct motor output (i.e., no significant muscle responses detectable via electromyography) but play an essential role in motor programming and control.

These achievements are largely grounded on the analysis of the reported data obtained from experiments on non-human primates, which serve as the most reliable animal model for inferring knowledge on the human nervous system. In this narrative review, we explore the translational impact of studies on non-human primates (used as a reference frame) on the development and refinement of technologies and techniques in clinical neurosurgery. In addition, we provide insights for future perspectives.

## 2 The functional organization of circuits subserving motor abilities: lessons from non-human primates

Jackson (1864) declared that he could find *“no more difficulty in supposing that there are certain convolutions superintending those delicate movements of the hands which are under the immediate control of the mind, than that there is one, as Broca suggests, for movements of the tongue in purely mental operations”* (Penfield and Boldrey, 1937), thus suggesting the existence of a cortical area devoted to movement and muscle control. Jackson’s intuition was confirmed in 1870 when Hitzig and Fritsch first evoked movements through electrical stimulation in animal models of an area of the neocortex now known as the *motor cortex* (Fritsch and Hitzig, 2009). The first scientific report of stimulation of the motor cortex in humans was provided during the same year by an American surgeon, Bartholow (1874). Despite more than a century having passed since these illuminated observations, the characterization of the anatomo-functional distinguishing features of cortical areas involved in the control of motor abilities in humans still represents a challenging matter.

### 2.1 The Corticospinal system and voluntary movement

“A number of evolutionary processes together resulted in a purposefully use of the hand and arm under the dominant control of the cerebral cortex” (Sherrington, 1906). According to the electrophysiological studies of Leyton and Sherrington (1917) on primates and of Penfield and Boldrey (1937) on humans, the area of the neocortex hosting the executive control of skeletal muscle activation was constrained to the circumvolution anterior to the central sulcus, i.e., the precentral gyrus. This area is now addressed as the primary motor cortex (M1). Located anterior to the M1 is the premotor cortex (PM), originally considered to be a functionally distinct area more related to motor programming of complex voluntary movements rather than responsible for their actual execution (Awalter, 1905; Farquhar, 1935; Jacobsen, 1934; Jacobsen, 1935). However, a clear motor output evoked through direct cortical stimulation was also observed by stimulation of the PM (Farquhar, 1935; Penfield, 1951; Woolsey et al., 1952; Wiesendanger, 1981). It took over a century to provide evidence that motor output observed by these motor areas was due to a system of descending fibers ultimately acting on spinal motoneurons and, importantly, that the control of voluntary movement is far more complex and not limited to the mere activation of skeletal muscles. The control of the spinal cord machinery required for voluntary movements is mediated by a system of fibers forming the corticospinal tract (CST). These fibers originate not only from the M1, which contributes only a small percentage, but also from a complex mosaic of frontal and parietal areas. These regions are densely interconnected, allowing for a coordinated descending control of the lower components of the motor system (Dum, 1991).

The synaptic architecture and the functional properties of the CST have been investigated extensively in non-human primates, which represent the best animal model to study motor control since they share with humans the same level of dexterity. Retrograde transneuronal transport of tracers from single muscles in non-human primates showed that within the frontal lobe, only 50.9% of the CST fibers originate from the M1, and the remaining 49.1% originate from the PM areas outside of the M1 and, in particular, the dorsal premotor cortex (PMd—18.9% of total), supplementary motor area (SMA—12.4% of total), dorsal cingulate motor area (CMAd—8.3% of total), ventral premotor (PMv—4.4% of total), ventral cingulate motor area (CMAv—2.7% of total), and rostral cingulate motor area (CMAr—2.3% of total) (Dum and Strick, 2005). Aside from the frontal lobe, the parietal lobe contributes with a relevant component of fibers from the primary somatosensory cortex (S1) and the posterior parietal cortex.

Crucial to understanding the role of the different areas in CST action on spinal cord machinery is the knowledge of their pattern of termination. Kuypers et al. showed that corticospinal neurons located in the precentral gyrus project mostly contralaterally—via the lateral corticospinal tract—to the dorsolateral region of the intermediate zone of the spinal gray matter hosting the interneurons acting in modulating the excitability of the dorsolateral motoneuronal pools innervating distal muscles (Kuypers et al., 1962). Within the lateral CST, the neurons originating from the M1 project directly to the laminae hosting motoneurons controlling the muscles of the extremity: the hand and digit (Kuypers et al., 1962; Lemon, 2008). Some corticospinal fibers descend ipsilaterally in the spinal cord—via the ventral corticospinal tract—to target bilaterally the ventromedial intermediate zone, controlling trunk and girdle muscles. Despite the similarities in the pattern of termination of CST fibers from different frontal cortical areas (He et al., 1993), relevant differences in the projections among them have been found (Maier, 2002; Boudrias et al., 2006). CST projections arising from premotor areas are organized in a topographic fashion independent from the somatotopic representation of the M1, with the number of CS neurons arising from the PMd covering a larger cortical area compared to the PMv’s CS neurons, which are clustered within a limited area. The CST fibers originating from the postcentral gyrus (namely S1) terminate mostly in the dorsal horn and avoid the rest of the spinal gray matter.

The difference in their relative contribution to the CST, together with their different pattern of termination in the spinal cord laminae and segments, suggested that the different CST areas may subserve different but complementary roles in motor control.

Starting from this evidence, the aspect to be investigated regarding the motor cortex was the actual topographic organization of motor output. The use of intracortical microstimulation (ICMS) coupled with neuronal labeling with tracers of the stimulated areas on Cebus monkeys found the largest digit and arm distinct representation in the PMd, PMv, and SMA (He et al., 1993; Rizzolatti et al., 1988; Hepp-Reymond et al., 1994; Godschalk et al., 1995; Dum and Strick, 2002; Cerri et al., 2003; Raos et al., 2004). All these areas were densely interconnected both among themselves and with the M1 (Dum and Strick, 2005).

Particularly interesting is the output from the M1 that, when lesioned, leads to permanent deficits. The idea that discrete cortical areas control single muscles suggested by Penfield and Boldrey (1937) and Leyton and Sherrington (1917) was challenged by ICMS data in non-human primates, reporting contradictory findings about the origin of the motor output from the M1. Asanuma et al., for instance, described a “*finely grained mosaic (of single muscle representation) within the depth of M1*” (Asanuma and Rosén, 1972), while Phillips and Porter (1977) found that effectors for hand movements were distributed on broad areas of M1 and were widely overlapped. Of notable interest in shedding light on this issue is the pattern of origin and termination of the direct projection to motoneurons (corticomotoneuronal—CM neurons), which correlates with the emergence of dexterity. CM cells are primarily located in the central sulcus, over a broad medio-lateral region classically identified as the *arm area*. The observation that CMs targeting different hand muscles are widespread over the arm area, overlapping with the CM for shoulder muscles, suggests that the motor output is organized in terms of muscle synergies, rather than with a single muscle rationale (Rathelot and Strick, 2009; Rathelot and Strick, 2006). Notably, CM neurons were found predominantly within the caudal area of the M1, highly excitable with the lowest threshold for motor responses in digits, elbow, and shoulder (Dum and Strick, 2005), while the rostral portion of the precentral gyrus hosts also fibers directed to the red nucleus (cortico-rubro projections). In light of this evidence, the new concept of the anatomo-functional organization of the primary motor area in two subsectors called “*new”* and *“old M1*” emerged. The *old M1*, located in the rostral area of the motor cortex on the convexity surface of the precentral gyrus, hosts neurons ultimately projecting to interneurons of the spinal cord, acting on *α*-motoneurons through an indirect pathway. On the other hand, the *new M1,* located caudally and constrained in the sulcal surface of the precentral gyrus, is the area mainly hosting CM neurons and reaching the *α*-motoneurons directly. The CM neurons were found also in the *old M1*, although with slower conduction velocity and lower amplitude of post-synaptic potentials compared to the CM cells found in the *new M1*. Finally, supporting this functional subdivision of the M1, different connectivity of rostral and caudal M1 has been reported, the former being more densely connected to premotor areas, whereas the latter being more tightly connected to parietal sensory areas (Stepniewska et al., 1993; Gould et al., 1986; Dancause et al., 2007; Dea et al., 2016). All these data pointed to the conclusion that the *old and new* M1 (Dum and Strick, 2005; Strick et al., 2021) belong to different cortical networks within the CST, thus exerting a different function in motor control (Stepniewska et al., 1993; Gould et al., 1986; Dancause et al., 2007; Dea et al., 2016).

Ultimately, some direct CM cells (15%) were found in area 3a, classically considered part of the primary sensory area (S1). These neurons, terminating on the dorsal horn, do not exhibit a motor output (Widener and Cheney, 1997) but rather could act in gating sensory inputs to the spinal cord (Lemon, 2008).

### 2.2 The human primary motor cortex and the *hand knob*

Given the evidence obtained with non-human primates, a heterogeneous organization of the motor cortex, in particular of the *hand knob*, was expected in humans as well. The first reports in line with this view came from functional imaging (Kawashima et al., 1995) and cytoarchitectonic (Geyer et al., 1996) studies. Two different areas with increased regional cerebral blood flow during hand and finger movements were found: one located in the anterior lip of the central sulcus and the other relatively close to the surface of the precentral gyrus (Kawashima et al., 1995). Geyer et al. (1996) based on the observation of a different distribution of pyramidal neurons in layer III of the precentral cortex together with the different distribution of serotoninergic neurotransmitters binding sites, disclosed two distinct areas within M1, called area 4a, the anterior M1, and 4p, the posterior M1, possibly recalling the *rostral* and *caudal* subdivision of M1 in non-human primates. Further non-invasive fMRI studies analyzed the neural activation of the M1 in subjects performing stereotyped movements while gradually changing the amount of attention required for the task (Binkofski et al., 2002). In line with previous studies (Geyer et al., 1996), they found a significant difference between the two regions: a region matching with the Brodmann area 4p localized in the anterior bank of the central sulcus, actually modulated by attention, and a region matching with the Brodmann area 4a in the posterior bank of the precentral gyrus, not significantly modulated by the attentional visual cue. Despite being very interesting, these data are limited by the accuracy of fMRI studies in investigating M1 somatotopy failing to distinguish two hand representations (Binkofski et al., 2002; Meier et al., 2008), thus preventing these studies from being conclusive. To have robust evidence in favor of the subdivision of M1, studies directly stimulating human M1 are mandatory. However, direct access to the human cortex is exceedingly rare, as evidenced by the limited literature available on the subject, the majority of which is derived from data collected during brain tumor surgeries utilizing brain mapping techniques (detailed in the next sections).

## 3 Intraoperative stimulation techniques of the human cerebral cortex

The *brain mapping technique* used to guide the resection of intrinsic brain tumors—aimed at preserving essential structures within circuits that control motor abilities—relies on two protocols of stimulation: high-frequency direct electrical stimulation (HF-DES) and low-frequency direct electrical stimulation (LF-DES). When approaching tumors involving motor areas, commonly considered to be located in the frontal lobe close to the M1, the identification of the areas essential for preserving voluntary movement has historically relied on the detection of motor responses to electrical stimulation, which indicates the connectivity of the stimulated area with the spinal motoneurons.

The LF-DES technique delivers long trains (1–4 s) of biphasic pulses at low frequencies (50–60 Hz). The motor output can be monitored either with visual inspection or with free-running EMG (electromyography) (see Table 1). LF-DES mapping is generally performed in awake conditions for two main reasons: The current required for eliciting a motor response is lower, thus abating the risk of seizures; the stimulation paradigm is more efficient when the patient is awake, thus reducing the risk of negative mapping.

The HF-DES protocol delivers current in short high-frequency (250–500 Hz) square wave trains and monophasic pulses with a low-train repetition rate (0.5–2 Hz) (see Table 1). This technique is commonly used with a fixed protocol of five pulses, referred to as the “train-of-five” technique (To5), to stimulate motor areas aimed at eliciting a motor output, the motor-evoked potentials (MEPs), to be recorded using the EMG of the patients’ muscles.

Over the past two decades, these two paradigms have been developed based on knowledge acquired from animal studies (Dum, 1991; He et al., 1993; Rathelot and Strick, 2009; Rathelot and Strick, 2006; He et al., 1995), enabling the mapping of different areas and functions, and allowing their application in various clinical contexts (see Table 1). This effort represented a significant step forward in the treatment of brain tumors.

### 3.1 Low-frequency stimulation mapping motor areas: the motor homunculus as a reference frame

Historically, brain mapping of the motor areas was performed with LF-DES, based on the original Penfield’s paradigm (named then as “faradic” stimulation), a technique used since the beginning of the 20th century (Penfield and Boldrey, 1937), thus considered to be safe and familiar to most neurosurgeons all over the world. Responses to LF-DES are commonly detected only via visual inspection—rare is a surgical setting equipped with free-running EMG recording to monitor the excitation of muscles—and the surgery relies, as a main reference frame, on the actual topographic organization of motor output, originally described by Sherrington, Woolsey, and, in humans, Penfield as the “*sensorimotor homunculus*” (Penfield and Boldrey, 1937).

In 1937, Penfield systematically stimulated the precentral gyrus in surgical patients in awake conditions. Based on the motor responses observed, he disclosed the well-known “sensorimotor homunculus,” the graphical representation that simplifies the representation of muscle effectors on the surface of the cerebral cortex taking into account the area of the cortex hosting the maps of responses from the toes, legs, trunk, arm, digits, hand, face, eyes, mouth, and tongue. The homunculus that emerged from the anatomical transposition of the motor responses was caricatural, disclosing the fundamental concept [confirming non-human primates’ data (Penfield and Boldrey, 1937)] that the cortical areas hosting the different body parts are not faithful to their real dimensions, but rather to their functional relevance in the motor repertoire. As a result, the face and hands are far bigger than the lower limb or trunk. Interestingly, although the original stimulation maps revealed cortical sites responsive to different body segments, including multiple sites for the same segment, the overlap among effectors’ motor responses was overlooked when these data were converted into a visual representation. This resulted in the loss of notable data pointing to a body representation with intermingling, rather than discrete, somatotopy of body segments on the primary motor cortex. Thus, the *motor homunculus* has been a reference frame for surgeons during the resection of brain tumors with LF-DES, despite its substantial limitations. First, Penfield and Boldrey’s study (Penfield and Boldrey, 1937; Asanuma and Rosén, 1972) was based on qualitative rather than quantitative data, in the absence of a quantitative electromyography (EMG) recording. Studies performed in the last decade clearly showed that the information inferred from the EMG recording is far more precise and allows the investigation of the fingerprints of the functional properties of the stimulated structures compared to the sole visual inspection of the elicited movement. Second, due to the lack of technological support, the stimulation sites were empirically reported on a chart for every single patient from a sketch or a picture of the operatory field—using as main reference the distance to the Sylvian and the median longitudinal fissures—and then transferred on a common final chart for all the patients, an unauthorized operation given the interindividual differences in brain anatomy. Moreover, the representation of the lower limb, based on scarce data due to the limited access to the mesial surface of the precentral and postcentral gyrus, was not reliable.

Despite this limitation, Penfield’s model remained unchallenged for almost a century, until a similar experiment was conducted by Roux et al. (2020), stimulating with biphasic square pulses (1 ms duration, each phase 0.5 ms) in 50 Hz trains (max duration 3 s) awake patients during surgery for brain tumors, again in the absence of EMG recording. The main difference compared to Penfield’s findings was that LF-DES always elicited stereotyped movements failing to elicit “ecological” synergies. Despite this difference, the common emerging feature was the relative somatotopy with a medial-to-lateral organization of body parts representations: In both studies, digit movements were reported for stimulation of the lateral part of the hand regions, the most common response being the flexion of all fingers, reported by Penfield as “*an individual movement, not a combination of separate movements with individual representation*.” Overall, Roux et al. described a somatotopy (intended as a point-to-point correspondence between an area of the cortex and a specific body part movement) along a medial-to-lateral and a *fine-grained* distribution. Notably, according to their reports, movements of body segments were not constrained to the stimulation of small/focal area of the cortex, but they could be evoked by multiple sites on the precentral gyrus, intermingling with other agonists on the same area, in line with what observed on primates (Asanuma and Rosén, 1972). Thus, as discussed above, whereas the idea of the homunculus is overall correct in identifying different body areas (e.g., hand or face), a rigid distinction between body parts divided by sharp borders over the cortex stimulation is no longer acceptable.

Given its translational impact in the neurosurgical setting for brain tumor removal, the concept of the sensorimotor homunculus is still a matter of debate and it must be carefully considered when relying on it during surgery. During the removal of brain tumors harboring within the frontal motor areas, surgeons stimulate directly cortical and subcortical structures to identify responsive essential sites to be preserved. This unique setting, allowing direct access to the human brain, can be considered the neurophysiological setting closer to the ICMS used in non-human primates described above to investigate the organization and somatotopy of the motor cortex (Dum, 1991; He et al., 1993; Asanuma and Rosén, 1972; He et al., 1995; Kwan et al., 1978). However, considering the ICMS stimulation is far more refined and controlled and due to the relevant differences between the macaques’ and humans’ brains, the attempt to fit human data in the non-human primates’ frame remains a challenge to be faced by acquiring data in the human setting to be matched with non-human data to build a conclusive human map.

### 3.2 High-frequency stimulation: efficacy of synaptic temporal summation on CST fibers

HF-DES is a technique recently introduced to monitor motor-evoked potentials (MEPs) under general anesthesia (Taniguchi et al., 1993), which is a condition that, by reducing the excitability, decreases the efficacy of LF-DES to elicit motor responses. Taniguchi et al. first proposed the stimulation of the human cortex through a monopolar probe and high-frequency trains of stimuli in 1993 as a monitoring method. This technique (trains of 2–9 monophasic anodic pulses, typically 5) at 500 Hz grounds on non-human and human primate studies.

The first reports of the use of train of short pulses of anodal stimulation of the motor cortex date back to the studies of Hern et al. (1962) on African baboons (Papio species) reporting that surface-anodal square pulses selectively stimulated the corticofugal neurons. Later studies confirmed these findings (Taniguchi et al., 1993; Cedzich et al., 1996; Kombos et al., 1999). The use of short trains of pulses at high frequency (i.e., with short interstimulus interval) had already been reported on animals (Asanuma and Rosén, 1972) and on a small number of non-anesthetized patients as described by Milner-Brown et al. (1975). In 1993, Taniguchi et al. suggested a protocol to monitor motor function during surgery to be adopted as an alternative to LF-DES bipolar stimulation. In their study, they delivered short trains of one to five rectangular pulses (200 to 500 μ sec each) with an interstimulus interval varying from 1.25 and 5 ms on nine patients operated under general anesthesia equipped with EMG recording (Taniguchi et al., 1993). They found that the lowest current intensity of stimulation to elicit a reliable motor response (motor-evoked potentials, MEPs) in muscles (motor threshold) varied depending on the number and duration of pulses as well as on the location of the electrode and that the amplitude of muscle responses correlated with the intensity of stimulation, the number of pulses, and the duration of each pulse. In line with the studies of Gordon et al. (2023), anodal stimulation of the cortex was more efficient compared to the cathodal. The efficacy of short trains of rectangular anodal pulses is based on the assumption that the excitability of corticofugal neurons under general anesthesia, compared to awake conditions, impacts the responsiveness to DES as shown by recording, with epi- or subdural electrodes in the human spinal cord, the descending volleys elicited by the stimulation of the motor cortex and conducted by the CST. CST volleys can be recorded as a complex composed of the direct (D) wave and the indirect (I) waves: The D-wave represents synchronous activity of the fast-conducting CST neurons directly activated by the current either distally or near the cell bodies, while the I-waves represent the trans-synaptic activation of CST neurons, some of which already directly activated (and represented in the D-wave). The summation of the effect of the subsequent descending volleys ultimately drives the membrane potential of spinal *α*-motoneurons to the firing threshold thus activating skeletal muscles, originating the MEPs. In awake condition, DES delivered on the cortex elicits both D- and I-waves exciting motoneurons, whereas, given the susceptibility of the I-waves to anesthetic drugs, with the same intensity in asleep conditions, only the D-wave is evoked, explaining the need of a higher intensity to elicit MEPs in this condition. Short trains of pulses delivered at a high frequency, by means of the temporal summation of post-synaptic potentials, elicit both I- and D-waves, allowing to reach the *α*-motoneurons’ threshold generating MEPs even under general anesthesia. Although this technique seems to evoke only 5% of the total motor units in the target muscle, it has to be considered comparable to other techniques of motor monitoring, such as the conventional transcranial electrical stimulation.

High-frequency stimulation was therefore found to be suitable to fire CST fibers in that, by taking advantage of the temporal summation of EPSPs in the post-synaptic membrane succeed to activate resting CST neurons despite the lower excitability, avoiding to increase the intensity of stimulation which could result, as side effect in seizures and lack of focality of stimulation. The potential of this technique has been exploited by tailoring the HF-DES protocol to the electrical properties of the different sector of the motor cortex, achieving relevant results in fostering tumor resection, as detailed in the following sections (Rossi et al., 2021a; Rossi et al., 2020).

## 4 Efficacy of HF-DES and LF-DES combination in enhancing resection of motor lesions: the turning point

The analysis of the LF- and HF-DES protocols suggests that both techniques can be used in brain mapping. However, given their distinguishing features in the activation of the neural elements, they cannot be considered interchangeable but should be tailored to the properties of the structures to be identified and to the clinical context. This is the main principle grounding the first study conducted by Bello et al. (2014) aimed at comparing the use of HF-DES and LF-DES in a large cohort of brain tumor patients (591 patients). Since then, the gold standard for motor mapping was LF-DES, either in awake or asleep conditions (Ojemann and Whitaker, 1978; Berger, 1995; Duffau et al., 2003; Keles et al., 2004; Yamaguchi et al., 2009), and only a few reports were available on the possible alternative use of HF-DES to map motor output (Szelényi et al., 2011; Cedzich et al., 1996; Kombos et al., 1999; Bello et al., 2007; Bello et al., 2008) which seemed to be necessary given the limitations of LF-DES (see Paragraph 4.1), such as low effectiveness in general anesthesia and a high risk of inducing seizures.

Bello et al. (2014) performed an intra-individual analysis of the effect of HF- and LF-DES when approaching tumors involving the CST areas/pathways of the frontal lobe. In a group of patients, cortical and subcortical mapping was performed starting with the gold standard LF-DES and then switched to HF-DES when it failed to elicit motor responses. A group of patients treated with the sole LF-DES was used as the control group. The analysis outlined different sets of patients, clustered based on their clinical features emerging as relevant determinants of the effectiveness of one or the other of the techniques. LF-DES protocol was effective in patients with a short seizure history, a maximum of 2 AEDs intake, and well-defined masses on FLAIR images. However, HF-DES protocol was needed to obtain a higher extent of resection, while minimizing the risk of permanent motor deficits, in patients with a longer seizure history, a higher number of AED intakes, diffuse tumor margins on FLAIR sequences, frequently displaced or infiltrated CST, and previous oncological treatments. These clinical and radiological characteristics, taken together, defined a population of patients that was considered at “higher risk” since the tumor was likely altering the excitability of the CST that hence required the application of HF-DES paradigm for motor mapping. The analysis of the control group supported the results. This study set the turning point in preserving motor functions in the surgery of brain tumors in that it clarified a relevant concept: Brain mapping protocols must not be applied with a rigid rationale for choosing one or the other protocol, rather they need to be both available and it is the “flexible” combination of the two techniques, strongly depending on the clinical context, that increases the efficacy and overcomes the limitations of each. In particular, HF-DES, with its short trains and its low incidence of seizures, succeeds in eliciting motor responses in clinical conditions characterized by a long history of seizures, and tumors infiltrating the CST, not approachable by LF-DES. As stated above, short trains of multiple pulses very efficiently excite the CST fibers, even in unfavorable conditions, such as fibers damaged by neoplastic disease, a long history of seizures, or during general anesthesia (thus not requiring awake conditions). HF-DES elicits MEPs that are monitored through EMG recording, which allows the detection of the earliest signs of muscle responses to stimulation, i.e., the excitation of few motor units and thus preventing further increase of current intensity (raising the risk of seizures) to generate overt clear movement of the body segment needed for the intraoperative visual inspection. This can be considered as a relevant advantage and a limitation at the same time: To perform an intraoperatory EMG, multi-channel EMG machines are needed, and they are not always available in neurosurgical units; moreover, an EMG must be interpreted in real time by an experienced neurophysiologist or a neurophysiology technician.

In addition, HF-DES elicits MEPs with a similar morphology at any level of the CST (both cortical and subcortical), as opposed to LF-DES, which recruits motor units progressively when delivered to the cortex, but its effect fades away subcortically (Szelényi et al., 2011). Moreover, motor responses elicited by LF-DES at a subcortical level are impaired by the use of ultrasonic aspirators, a diffuse tool for tumor removal, whereas HF responses are not affected (Szelényi et al., 2011).

As an overall result, when HF-DES is available, the extent of resection is increased, and the amount of permanent neurological deficit is reduced (Bello et al., 2014).

In addition to the technical issues related to the efficacy of the two protocols in exciting neural elements, the decision on the technique to be adopted must take into account the intrinsic properties of the areas and tracts to be stimulated during brain mapping. When approaching the corticospinal system, the mapping must be grounded on the available knowledge of its anatomo-functional properties. CST originates from a mosaic of areas from the frontal to the parietal lobe. Non-human primate studies revealed different functional properties of the different CST areas, suggesting that the excitability of neural elements cannot be considered homogeneous among areas (Rathelot and Strick, 2009; Rathelot and Strick, 2006). Therefore, it seems reasonable to expect different neurophysiological properties in the motor areas in humans as well, hence the need for different paradigms to correctly elicit a motor response. Whereas Bello et al. provided evidence that HF-DES is efficient in eliciting responses from the primary motor cortex (M1), non-primary motor areas (SMA and PM), and even sensory areas (S1, although it is not clear if due to current spread) (Fornia et al., 2018; Bello et al., 2014), the efficacy of this protocol in differentially identifying these areas and correctly mapping them was not reported. It took the next decade (2014–2024) to design and implement adequate protocols guiding brain mapping of the different CST areas (see following Paragraphs 6 and 7) to preserve motor abilities from dexterity to praxis.

## 5 Development of HF-DES technique enhanced resection of lesions in the primary motor cortex

In the particular setting of tumors involving motor structures (M1 and descending fibers), resection of the tumor while sparing the motor function is challenging. The classical model of cortical and fibers organization, as drawn by Penfield in 1937, has been partially dismantled by the cytoarchitectonic analyses, anatomical tracers, and ICMS applied to non-human primates. This set the ground for the hypotheses of a more complex organization of M1 and in particular of the hand area (Rathelot and Strick, 2009; Dea et al., 2016; Witham et al., 2016) revealing two functional components: fast-conducting cortical neurons, located in the caudal part of the precentral gyrus and anterior bank of the central sulcus; slow conducting neurons, originating from the rostral M1, located on the anterior portion of the precentral gyrus. In humans, the M1 hand area is denominated “*hand knob*,” due to the characteristic appearance as an omega or epsilon-shaped bulge on axial MR images. Investigations in humans suggested an organization along a rostrocaudal gradient similar to the non-human primates (Geyer et al., 1996; Binkofski et al., 2002; Bastiani et al., 2016; Glasser et al., 2016; Amiez and Petrides, 2018). An analysis of this human vs. non-human homology in M1 anatomo-functional organization was proposed by a study integrating direct cortical stimulation in a neurosurgical setting and functional imaging (Viganò et al., 2019), showing higher excitability of the caudal part of human M1 cortex compared to the rostral portion. When HF-DES is applied to M1, a lower number of pulses and lower intensities are needed to evoke responses from the caudal portion compared to the rostral. These data could support and point to a functional subdivision of M1 in a rostral and caudal sector recalling the *old* and *new* non-human primates’ M1 areas: the rostral M1 in humans as the origin of fibers indirectly acting on spinal *α*-motoneurons, therefore less excitable, and the caudal M1 with fast direct corticomotoneuronal fibers, requiring a lower intensity to elicit responses. However, another possible explanation is that direct cortical stimulation of the rostral precentral gyrus elicits responses by exciting the neurons on the anterior bank of the precentral gyrus, thus requiring higher intensities to reach a deeper distance. Alternatively, the rostral M1 could be a transitional area between M1 and the dorsal premotor cortex (Viganò et al., 2019).

In this respect, interesting is the effect of LF-DES applied on rostral and caudal M1 in awake patients while performing a specific motor task. Regardless of the specific design of the task, which will be explained extensively in the next paragraph, notable is the significant difference in the effect of the stimulation on the muscle activity in the two sectors, not expected should the rostral and caudal M1 be identical. LF-DES on the rostral hand knob interferes with the task either by suppressing the ongoing muscle activity or by changing the pattern of muscle synergies activated, resulting in a dysfunctional activation, while stimulation of the caudal hand knob causes progressive recruitment of hand and forearm muscles along with the increase of stimulation intensity. Interestingly, both the behavioral outcomes associated with LF-DES on rostral M1 were associated with forearm and proximal muscle recruitment. This evidence was in line with previous reports on monkeys, showing that a central core of distal muscle representation is surrounded by distal and proximal muscles overlapping and by a horseshoe-shaped proximal muscle zone (Park et al., 2004; Hudson et al., 2017). Discussion of these data suggests that the rostral hand knob might play a role in the implementation of functional synergies between distal and proximal muscles during upper limb multi-joint movement (Viganò et al., 2019). Another possible interpretation grounds on the analogy of this area with area F2 of non-human dPM since the latter has been shown to have both distal and proximal muscle representation (Dum and Strick, 2005; Boudrias et al., 2010). Distinct intracortical connectivity of the two areas, with rostral M1 connected through fronto-frontal U-shaped fibers to the superior and middle frontal gyrus, and the caudal M1 connected through fronto-parietal U-shaped tracts to the postcentral gyrus, also reported (Viganò et al., 2019).

**Figure 1:**
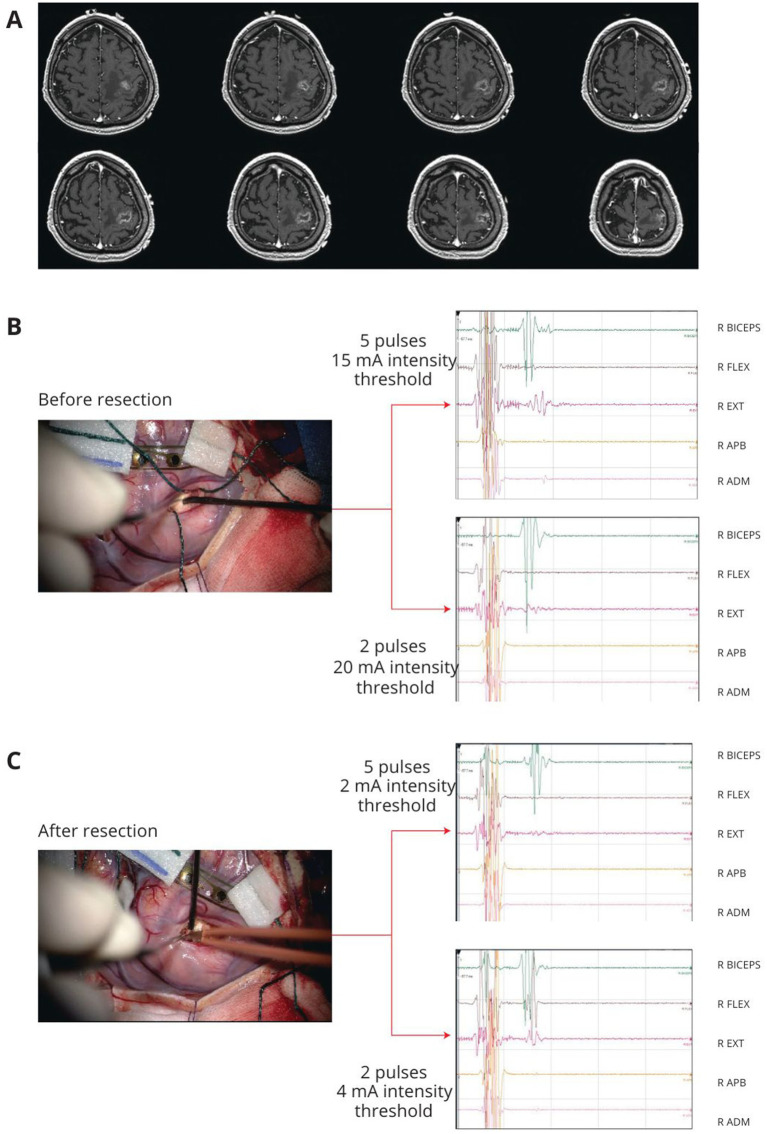
Example of left precentral tumor. After cortical grid placement on the M1, the precentral gyrus was mapped with HF-DES under general anesthesia. **(A)** T1 weighted post-contrast enhancement MRI of a patient with a left frontal lesion involving the precentral gyrus. **(B)** Before lesion resection, subcortical mapping with HF-DES was first performed with a 5-pulse stimulus, reaching an intensity threshold for MEP stimulation at 10 mA. At the same site, a 2-pulse stimulus was applied, reaching an intensity threshold for MEP stimulation at 15 mA. **(C)** Subcortical mapping with HF-DES was applied after resection, on the same simulation site shown in B. With a 5-pulse stimulus, an intensity threshold to evoke MEP was reached at 2 mA, whereas with 2-pulse stimulation, the intensity threshold was 4 mA, allowing extension of the resection to the limit of the lesion. HF-DES, high-frequency direct electrical stimulation; MEP, motor-evoked potential (Written informed consent was given by patients for image acquisition and publication).

The important translational impact of data provided in non-human primates first and then in humans on the functional subdivision of M1 is the expected different susceptibility of the two subsectors to the different DES protocols in that efficacy of the current intrinsically depends on the functional properties of the areas/tracts to be stimulated (as discussed in Paragraph 3.2). As expected indeed, the two M1 subsectors are characterized by different excitability, disclosed by the need to change the number and duration of pulses and the intensity of stimulation delivered to the rostral and caudal M1 during tumor resection to elicit motor responses (Viganò et al., 2019) leading in the implementation of a novel and efficient technique of excision of tumors harboring within M1, considered not resectable, through the use of HF stimulation described by the same group (Rossi et al., 2020) (see Figure 1). In this study, the authors outlined the use of different approaches based on stimulation parameters depending on the clinical condition. They described a *standard approach* consisting of a train of five pulses, with a duration of 500 μs and an interstimulus interval (ISI) of 2–3 ms. In this study, the standard approach was described as the first step in surgery, with the aim of understanding the cortical excitability: If it failed to evoke any response from the CST at the cortical level, the protocol was switched to an *increased train approach*. The *increased train approach* consisted of trains of 7–9 pulses, of a duration of 800 μs each (ISI 2–3 ms). This paradigm was efficient in evoking responses where the standard approach fails, such as the condition of a low cortical excitability. The standard approach was also used in combination with the *reduced train approach*, consisting of trains of two pulses, with a duration of 500 μs, very efficient when stimulating highly excitable tissue. The standard and *reduced* train (To2) combined approach were found to be effective in a context of high cortical excitability, where the standard To5 was unable to identify a safe (negative) entry zone on the cortex due to the low resolution of the stimulation electrical field. In this case, by reducing the number of stimuli, and switching to a train of two stimuli, only the fibers with very high excitability (since they need a lower number of pulses to be elicited) are selected, depicting them among the low excitability fibers, allowing to better define the cortical map of corticofugal fibers and therefore to determine the margins of corticectomy to access the tumor. The clinical application of the *reduced* train approach and its efficacy in brain tumor removal was extensively detailed in a comparative work by the same group (Rossi et al., 2021a).

First on the non-human primates studies reporting the subdivision of the primary motor cortex in old M1 and new M1, further supported in humans with DES studies (Viganò et al., 2019; Rathelot and Strick, 2009; Rathelot and Strick, 2006; Geyer et al., 1996; Binkofski et al., 2002) showing a gradient of excitability, being lower in PMd and gradually increasing to rostral area peaking in the caudal portion, Rossi et al. (2021a) investigated the responsiveness to the application of the standard and reduced approach of these two M1 sectors, to provide intraoperative tools to allow the distinction of these two components during motor area tumor resection (Figure 1). Interestingly, the results showed that not only HF with a To2 paradigm (HF-To2) is able to identify the fibers with the highest excitability but also that these connections proved to be:

- Faster: 23 ms latency compared to 25–26 ms of lower excitability fibers (Rossi et al., 2021a).
- Clustered on the dorsal portion of M1, in line with observations in non-human primates regarding the *new M1* area and the location of corticomotoneuronal fibers, or 4p in humans.
- Essential for fine motor movements: excision of the rostral portion of M1 and conservation of the dorsal area allowed to preserve the ability to recover from early postoperative motor deficits and to return to normal motor function.

In addition, the HF-To2 technique was able to disclose the architecture of M1 fibers both at a cortical and subcortical level, even in clinical settings where the physiological segregation of the two areas was completely distorted by tumor infiltration and fiber reorganization.

Notable is the clinical impact of the use of this *advanced motor mapping* technique, allowing to face M1 tumors, so far considered not resectable and to obtain a higher extent of resection, which is vital to grant a longer progression-free and overall survival, by reducing permanent deficits (Rossi et al., 2020).

## 6 Role of hMT and LF-DES in mapping tumors in premotor and parietal areas: motor function goes beyond the simple muscle contraction

The HF-DES approach has been proven to be efficient in most tumors infiltrating M1 and its fibers (Rossi et al., 2020), but its limitations emerge when used in the case of tumors involving non-primary motor areas or parietal areas: Sparing the primary motor area, it is not indeed sufficient to preserve the motor abilities. As reported by Rossi et al. (2019), a significant proportion of patients undergoing surgery under general anesthesia for tumors within a 2 cm distance from the central sulcus developed hand apraxia after surgery, despite the absence of new permanent motor deficits. Apraxia is a highly debilitating motor impairment that severely impacts the ability to perform highly skilled motor movements, which is difficult to rehabilitate and impacts the patients’ quality of life (Goldenberg and Spatt, 2009). This issue is grounded on the principle that motor abilities are supported by complex circuitries involving and connecting areas of CST and, in humans, extending beyond CST areas strictly considered to embed cognitive contents within the motor function (Fornia et al., 2024). Motor abilities, that span from dexterity to praxis, can be hypothesized assuming some degree of similarity between the human praxis network and non-human fronto-parietal circuits of grasping networks. PM and SMA together with S1 and SMG participate in the planning and execution of movements. In particular, non-human primates’ ventral PM areas play a role in hand–object interaction (Bonini et al., 2011; Rizzolatti et al., 2014), and dorsal PM seems to be involved in the control of arm movements for reaching and grasping actions (Raos et al., 2004; Johnson et al., 1996; Wise et al., 1997; Tanji and Hoshi, 2008). These areas are functionally connected in circuits subserving motor abilities by acting on the spinal cord machinery via CST and by acting on the primary motor cortex. In primates, neural mechanisms for controlling skilled hand actions primarily rely on sensorimotor transformations. Visuomotor and haptic transformations are mediated by circuits connecting specific inferior parietal with ventral premotor areas where sensory coding of objects’ features shapes the appropriate motor programs. In macaques, these parietal and premotor areas are nodes of a large-scale cortical network, designated as “lateral grasping network,” including specific temporal and prefrontal sectors involved in object recognition and executive functions, respectively (Fornia et al., 2024; Borra et al., 2017). Converging comparative evidence suggests that these circuits subserving transitive actions, such as object prehension and manipulation, may represent the *building blocks* from which the human praxis abilities have emerged. Human and non-human primates share similar parieto-frontal streams for controlling distinct, although complementary, aspects of the hand–object-oriented actions. Functional MRI (fMRI) studies showed that the hand-related parieto-frontal connectivity extends in humans to compose the so-called “praxis representation network” (PRN) (Króliczak and Frey, 2009). The human PRN is a large-scale, left-lateralized, temporo-parietal–frontal circuit supposedly involved in translating conceptual and sensorimotor information into purposeful hand-skilled acts (praxis), including transitive and intransitive hand gestures (Fornia et al., 2024).

**Figure 2:**
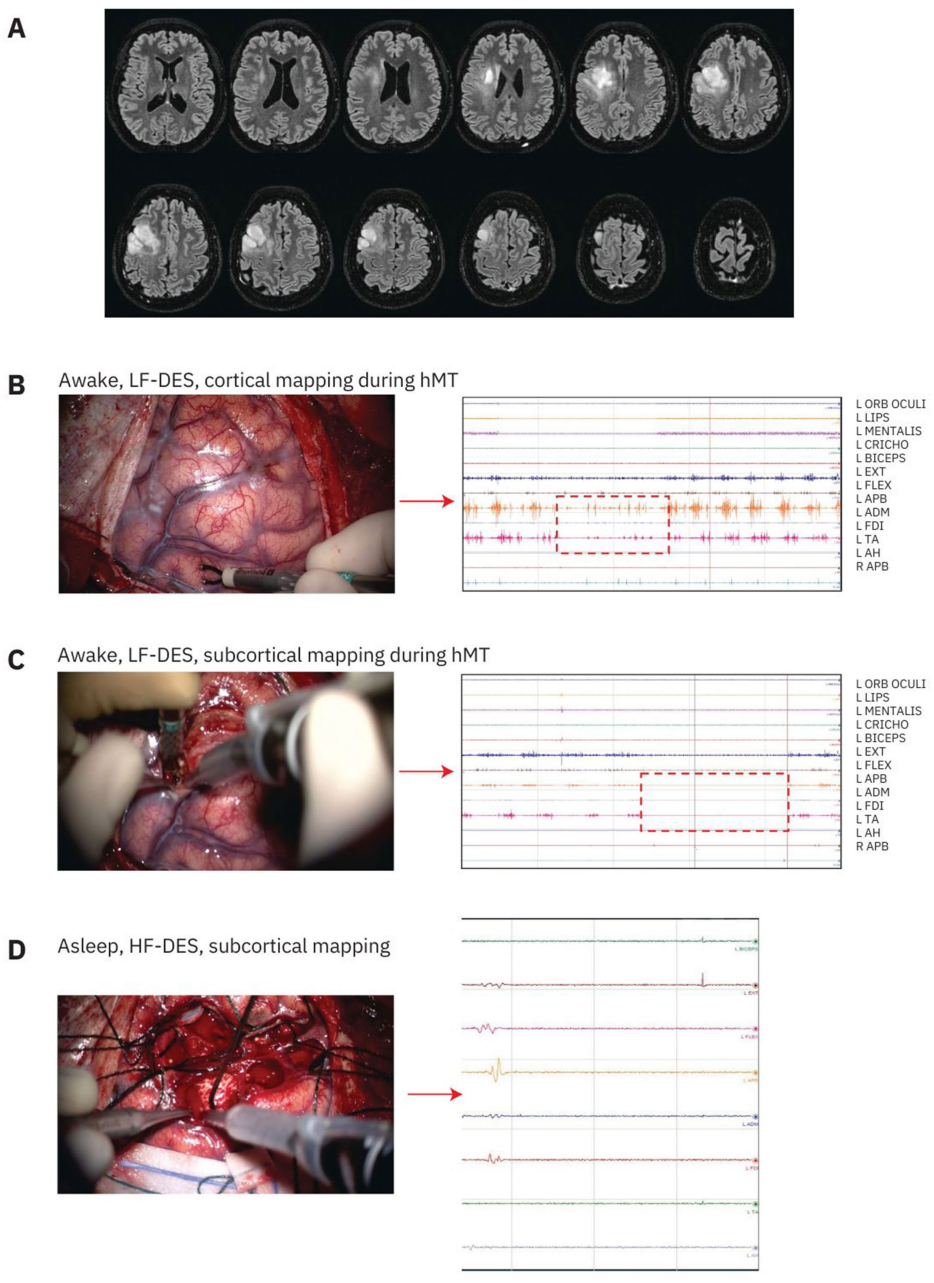
Example of right frontal tumor involving motor and premotor areas and relative mapping techniques. After cortical grid placement on the M1, the first phase consisted of an awake mapping with cortical LF-DES during hMT to identify the vPM and dPM. Afterward, corticectomy was performed and functional borders were defined through subcortical LF-DES during hMT. Finally, once functional margins were defined, resection was completed in general anesthesia with subcortical identification of the CST on the posterior border through HF-DES. **(A)** T2-FLAIR-weighted MRI of a patient with a right frontal lesion involving the precentral gyrus and premotor areas. **(B)** LF-DES stimulation with a bipolar probe in the awake setting during hMT. The figure shows an example of stimulation of the vPM inducing a disruption of the EMG recorded from left-hand muscles (APB, ADM, FDI), resulting in a block of the movement hand performed during hMT. **(C)** LF-DES in awake anesthesia performed during the definition of subcortical functional margins. Bipolar stimulation induced a complete block of muscle activity as reported on the EMG recorded from hand muscles (APB, ADM, and FDI). **(D)** Subcortical mapping of CST with HF in general anesthesia. The image shows the monopolar probe stimulation on the posterior border of the resection inducing MEPs in several contralateral upper arm muscles (biceps, extensor, flexor, APB, ADM, FDI; intensity threshold: 6 mA). LF-DES, low-frequency direct electrical stimulation; hMT, hand-manipulation task; vPM, ventral premotor; dPM, dorsal premotor; EMG, electromyography; APB, abductor pollicis brevis; ADM, abductor digiti minimi; FDI, first dorsal interosseous; CST, corticospinal tract; HF, high frequency; MEP, motor-evoked potential. (Written informed consent was given by patients for image acquisition and publication).

This overview of the literature highlights the importance of data recorded in non-human primates suggesting that while frontal areas are expected to generate a motor output, based on the specific pattern of termination, it is not reasonable to expect a motor output when stimulating parietal areas, even though they belong to the CST. Thus, the evolution of mapping over the last two decades has been based on the solid knowledge of non-human primate studies and progressively acquired useful data to understand homologies and differences with the human brain and shed light on the neural circuits underlying motor skills in a virtuous circuit of translational research. Grounding on the knowledge that a parieto-frontal grasping network has been suggested to exist in humans as well (Borra et al., 2017; Binkofski et al., 1998; Ehrsson et al., 2000; Ehrsson et al., 2001; Kantak et al., 2012; Geschwind, 1975; Nelissen and Vanduffel, 2011), involving parietal and frontal areas and connecting tracts, the brain mapping must be planned according to the different areas in different clinical contexts. Hence, while M1 and its descending fibers can be identified by monitoring the motor output (MEPs) elicited by DES without the need for the patient’s collaboration, structures involved in a higher level of motor programming and movement control (e.g., grasping networks) can be mapped only in the awake setting, while the patient is performing a motor task, based on the same principle used in brain mapping of language function. In this condition, mapping is not based on the identification of the *essential structures* based on its output; rather, the essential structure is identified based on the interference effect induced by DES while the patient is performing a task requiring the neurological function to be executed (Figure 2). When DES is applied on a brain structure actually involved in the circuit subserving the function to be assessed, current will disrupt the activity of the stimulated area thus leading to an impaired execution and an error is expected to occur and thus can be detected intraoperatively. In the specific neurosurgical context aimed at preserving motor abilities, the performance of a motor task has been so far the clinical routine for this purpose. The question is which is the adequate motor task to unravel different CST areas differently involved in motor control? To investigate non-primary motor areas, the commonly used intraoperative task consists of asking the patient to continuously perform a repetitive simple unpurposeful voluntary arm movement (flexion–extension) and observe the DES-induced interferences during task performance, allowing the identification of cortical or subcortical sites that, when stimulated, cause an interruption of the ongoing movement (Rech and Duffau, 2023). However, this task, requiring a simple flexion–extension, does not account for complex hand-manipulation abilities. Recently, the team of Prof Bello implemented a more advanced intraoperative test, called the hand-manipulation task (hMT) (Rossi et al., 2019) grounding on the non-human and human literature on the lateral grasping network. During this task, the patient sequentially grasped, held, rotated, and released the cylindrical handle continuously with the thumb and the index finger, using a precision grip. The proximity between the hand and the cylindrical handle allowed the patients to perform the movement using just the fingers, avoiding any reaching movements and thus requiring a hand–object interaction to be correctly performed. When DES is applied, interferences occur with the ongoing movement with different features when applied to different frontal and parietal cortical areas (S1, M1, vPM, dPM, aIPC—anterior intraparietal sulcus, aSMG—anterior supramarginal gyrus) or frontal white matter pathways, investigated in details by Fornia et al. (2020b), Viganò et al. (2019), and Viganò et al. (2022). The hMT, compared to the simple arm movement task, introduces the sensory-motor integration required for haptically driven hand–object manipulation, a crucial component of motor control of the task performance and thus supposed to detect the areas belonging to human praxis representational network (Fornia et al., 2024; Rossi et al., 2019; Borra et al., 2017; Lanzilotto et al., 2019). The analysis of the application of the two tasks (hMT or voluntary arm movement) in detecting sites in the white matter of the frontal or parietal lobe is at present not exhaustive, in that the full patterns of connectivity identified by DES during the execution of these tasks are not known (Rossi et al., 2021a). Notably, Rossi et al. (2019) reported that the clinical use of hMT dramatically reduces the incidence of postoperative ideomotor apraxia (global incidence of 2.5% when tasks are used compared to 50% when not applied) and the need for postoperative rehabilitation. This observation fostered further studies aimed at understanding the areas and the underlying connectivity involved in the praxis network. A lesion mapping study combined to direct stimulation during hMT recently showed that two sectors of parietal areas could be differently involved in praxis and sensorimotor integration (Fornia et al., 2024). In particular, two sectors within the parietal lobe seem to be involved differently in praxis functions: whereas resection involving inferior parietal areas seemed to be linked mainly to imitation of meaningful gestures, resection involving intraparietal areas affected both meaningless and meaningful gesture imitation, showing that more rostral areas seem to be involved in the visuomotor integration subserving the imitation of gestures, whereas inferior parietal areas are probably involved in connecting the praxis network to semantic knowledge. At the same time, intraoperative electrical stimulation of these areas during the hand-manipulation task-evoked different motor impairments confirmed the ability of this test to detect impairments of praxis functions.

## 7 Conclusion

Since the 7th decade of the 19th century, neuroscientists have been striving to unravel the organization of the human motor system. Major advancements in our current knowledge stem from experiments conducted on non-human primates, which are phylogenetically close to humans and share similar behavioral abilities, such as dexterous and goal-directed hand movements (Lemon, 2008; He et al., 1993; Rizzolatti et al., 1988; Dum and Strick, 2002; Cerri et al., 2003; Dum and Strick, 2005; He et al., 1995; Dum and Strick, 1991). As a result, our understanding of motor abilities and the neural circuitry underlying these functions continues to evolve.

Intrinsically connected to this research is the clinical context of brain tumor surgery, where the goal is to remove the tumor mass without impairing the patient’s functional integrity. To achieve this, DES is applied during surgery to identify critical structures based on their functional properties, ensuring they are preserved during tumor resection. The efficacy of brain mapping techniques relies heavily on our understanding of the neural circuitry underlying neurological functions, including the anatomo-functional properties of various neural structures (areas and tracts) and their specific roles in controlling particular functions. In particular, when addressing structures infiltrated by tumors that may be involved in motor abilities, brain mapping must be grounded in the continuously evolving scientific knowledge of the complex circuits that govern motor control. This growing body of research drives the need for implementing new intraoperative strategies, ultimately improving patient outcomes. Over the past decades, mapping strategies have undergone continuous refinement, increasing the likelihood of preserving full motor capacities.

Three key principles should guide the planning of brain mapping for preserving motor function: the clinical context of the tumor, the anatomo-functional knowledge of areas and tracts involved, and the availability of appropriate equipment and a multidisciplinary team to support the procedure.

Recent advances have highlighted the benefits of combining HF-DES and LF-DES in both asleep and awake conditions for mapping motor circuits. This approach improves tumor resection and patient outcomes, particularly for tumors involving motor pathways, and is now considered the gold standard.

Awake anesthesia, coupled with LF-DES mapping of premotor areas (notably vPM and dPM) and sensory areas, has proven particularly effective. By allowing patients to perform motor tasks such as the hand-manipulation task (hMT), this strategy enhances the identification of areas and circuits involved in praxis functions. It also optimizes the onco-functional balance, allowing for the resection of tumors in premotor and sensory areas while minimizing the risk of permanent motor deficits such as apraxia (Fornia et al., 2024; Rossi et al., 2019; Fornia et al., 2020a).

In contrast, HF-DES in asleep conditions remains the most effective approach for mapping the M1 and the CST at the cortical and subcortical levels, respectively. HF-DES can evoke motor potentials even under general anesthesia, enabling surgeons to estimate the distance to eloquent motor areas. Moreover, HF-DES can be tailored to the patient’s excitability, allowing for a personalized surgical approach based on individual characteristics. For tumors directly affecting the primary motor cortex or CST, three strategies are available: the standard approach, the increased train approach, and the reduced train approach (see Section 5 for details).

In summary, the mapping strategy should be carefully tailored to the clinical context, with the surgeon considering the networks involved and selecting the appropriate protocol based on the patient and tumor characteristics. While standard approaches using LF stimulation only require a stimulator and a trained anesthesiologist, advanced techniques involving HF stimulation demand state-of-the-art intraoperative technology. These techniques also require contributions from neuropsychologists, neurophysiologists, highly trained technicians, and neurosurgeons skilled in neurophysiology to accurately interpret evoked responses. This level of complexity introduces challenges related to costs and workflow volume (Rossi et al., 2021b).

Ultimately, the appropriate use of stimulation paradigms, anesthetic settings, and neuropsychological testing adapted to the patient’s unique characteristics is critical for accessing eloquent motor areas while preserving neurological function. These strategies enable maximum tumor resection, thereby improving progression-free and overall survival.

In conclusion, while significant progress has been made in treating brain tumors affecting motor circuits, much remains to be done, particularly in addressing circuits involved in phono-articulatory, cognitive functions, emotions, and mental states—areas not covered in this review. Nevertheless, basic research, especially studies conducted on non-human primates, remains a cornerstone for advancing clinical techniques. It is through this partnership between fundamental research and clinical practice that increasingly effective strategies are being developed to treat brain tumors, where surgery continues to be the most critical tool for disease control.
